# Supplementary material for: Pyruvate Dehydrogenase Kinase 1 inhibition mediated oxidative phosphorylation enhancement in cartilage promotes osteoarthritis progression
Source: BMC Musculoskelet Disord. 2023 Jul 20;24:597. doi: 10.1186/s12891-023-06585-6 (PMC10357736; doi:10.1186/s12891-023-06585-6)
Supplement: Supplementary file 1 — Additional file 1. [file 12891_2023_6585_MOESM1_ESM.zip › KEGG.Gsea.1653622432464/KEGG_RETINOL_METABOLISM.html]

Details for gene set KEGG\_RETINOL\_METABOLISM[GSEA]

|  || Dataset | 55457\_collapsed\_to\_symbols.cls.cls #OA\_versus\_normal.cls.cls #OA\_versus\_normal\_repos |
| Phenotype | cls.cls#OA\_versus\_normal\_repos |
| Upregulated in class | OA |
| GeneSet | KEGG\_RETINOL\_METABOLISM |
| Enrichment Score (ES) | 0.14805596 |
| Normalized Enrichment Score (NES) | 0.5852649 |
| Nominal p-value | 0.9948849 |
| FDR q-value | 1.0 |
| FWER p-Value | 1.0 |
Table: GSEA Results Summary

  

Fig 1: Enrichment plot: KEGG\_RETINOL\_METABOLISM      
 Profile of the Running ES Score & Positions of GeneSet Members on the Rank Ordered List

  

| SYMBOL | TITLE | RANK IN GENE LIST | RANK METRIC SCORE | RUNNING ES | CORE ENRICHMENT || 1 | PNPLA4 | patatin like phospholipase domain containing 4 [Source:HGNC Symbol;Acc:HGNC:24887] | 65 | 0.963 | 0.0739 | Yes |
| 2 | RPE65 | retinoid isomerohydrolase RPE65 [Source:HGNC Symbol;Acc:HGNC:10294] | 308 | 0.743 | 0.1161 | Yes |
| 3 | UGT2B4 | UDP glucuronosyltransferase family 2 member B4 [Source:HGNC Symbol;Acc:HGNC:12553] | 566 | 0.634 | 0.1481 | Yes |
| 4 | LRAT | lecithin retinol acyltransferase [Source:HGNC Symbol;Acc:HGNC:6685] | 1205 | 0.475 | 0.1374 | No |
| 5 | ALDH1A1 | aldehyde dehydrogenase 1 family member A1 [Source:HGNC Symbol;Acc:HGNC:402] | 1737 | 0.393 | 0.1283 | No |
| 6 | UGT2B17 | UDP glucuronosyltransferase family 2 member B17 [Source:HGNC Symbol;Acc:HGNC:12547] | 2050 | 0.356 | 0.1333 | No |
| 7 | DHRS4 | dehydrogenase/reductase 4 [Source:HGNC Symbol;Acc:HGNC:16985] | 2708 | 0.279 | 0.1050 | No |
| 8 | UGT2A3 | UDP glucuronosyltransferase family 2 member A3 [Source:HGNC Symbol;Acc:HGNC:28528] | 2854 | 0.265 | 0.1155 | No |
| 9 | BCO1 | beta-carotene oxygenase 1 [Source:HGNC Symbol;Acc:HGNC:13815] | 2895 | 0.261 | 0.1338 | No |
| 10 | CYP26A1 | cytochrome P450 family 26 subfamily A member 1 [Source:HGNC Symbol;Acc:HGNC:2603] | 3217 | 0.228 | 0.1276 | No |
| 11 | UGT1A8 | UDP glucuronosyltransferase family 1 member A8 [Source:HGNC Symbol;Acc:HGNC:12540] | 3301 | 0.221 | 0.1392 | No |
| 12 | DGAT1 | diacylglycerol O-acyltransferase 1 [Source:HGNC Symbol;Acc:HGNC:2843] | 3950 | 0.160 | 0.1020 | No |
| 13 | RDH11 | retinol dehydrogenase 11 [Source:HGNC Symbol;Acc:HGNC:17964] | 4102 | 0.146 | 0.1022 | No |
| 14 | CYP3A43 | cytochrome P450 family 3 subfamily A member 43 [Source:HGNC Symbol;Acc:HGNC:17450] | 4698 | 0.096 | 0.0638 | No |
| 15 | ADH5 | "alcohol dehydrogenase 5 (class III), chi polypeptide [Source:HGNC Symbol;Acc:HGNC:253]" | 4720 | 0.094 | 0.0699 | No |
| 16 | DHRS3 | dehydrogenase/reductase 3 [Source:HGNC Symbol;Acc:HGNC:17693] | 4770 | 0.089 | 0.0734 | No |
| 17 | UGT1A10 | UDP glucuronosyltransferase family 1 member A10 [Source:HGNC Symbol;Acc:HGNC:12531] | 4850 | 0.083 | 0.0741 | No |
| 18 | ADH1C | "alcohol dehydrogenase 1C (class I), gamma polypeptide [Source:HGNC Symbol;Acc:HGNC:251]" | 4908 | 0.079 | 0.0761 | No |
| 19 | RETSAT | retinol saturase [Source:HGNC Symbol;Acc:HGNC:25991] | 5356 | 0.043 | 0.0449 | No |
| 20 | RDH8 | retinol dehydrogenase 8 [Source:HGNC Symbol;Acc:HGNC:14423] | 5947 | -0.001 | -0.0009 | No |
| 21 | CYP2C19 | cytochrome P450 family 2 subfamily C member 19 [Source:HGNC Symbol;Acc:HGNC:2621] | 6089 | -0.011 | -0.0109 | No |
| 22 | CYP2C18 | cytochrome P450 family 2 subfamily C member 18 [Source:HGNC Symbol;Acc:HGNC:2620] | 6210 | -0.022 | -0.0185 | No |
| 23 | RDH5 | retinol dehydrogenase 5 [Source:HGNC Symbol;Acc:HGNC:9940] | 6277 | -0.026 | -0.0215 | No |
| 24 | UGT2B11 | UDP glucuronosyltransferase family 2 member B11 [Source:HGNC Symbol;Acc:HGNC:12545] | 7270 | -0.100 | -0.0905 | No |
| 25 | CYP2B6 | cytochrome P450 family 2 subfamily B member 6 [Source:HGNC Symbol;Acc:HGNC:2615] | 7445 | -0.114 | -0.0946 | No |
| 26 | DHRS9 | dehydrogenase/reductase 9 [Source:HGNC Symbol;Acc:HGNC:16888] | 7492 | -0.118 | -0.0886 | No |
| 27 | CYP2A6 | cytochrome P450 family 2 subfamily A member 6 [Source:HGNC Symbol;Acc:HGNC:2610] | 7849 | -0.144 | -0.1044 | No |
| 28 | CYP1A1 | cytochrome P450 family 1 subfamily A member 1 [Source:HGNC Symbol;Acc:HGNC:2595] | 8195 | -0.171 | -0.1173 | No |
| 29 | CYP3A5 | cytochrome P450 family 3 subfamily A member 5 [Source:HGNC Symbol;Acc:HGNC:2638] | 8272 | -0.177 | -0.1087 | No |
| 30 | CYP2C9 | cytochrome P450 family 2 subfamily C member 9 [Source:HGNC Symbol;Acc:HGNC:2623] | 8595 | -0.201 | -0.1173 | No |
| 31 | CYP4A11 | cytochrome P450 family 4 subfamily A member 11 [Source:HGNC Symbol;Acc:HGNC:2642] | 8654 | -0.205 | -0.1050 | No |
| 32 | ADH7 | "alcohol dehydrogenase 7 (class IV), mu or sigma polypeptide [Source:HGNC Symbol;Acc:HGNC:256]" | 8906 | -0.225 | -0.1061 | No |
| 33 | CYP3A7 | cytochrome P450 family 3 subfamily A member 7 [Source:HGNC Symbol;Acc:HGNC:2640] | 9024 | -0.234 | -0.0960 | No |
| 34 | ADH1B | "alcohol dehydrogenase 1B (class I), beta polypeptide [Source:HGNC Symbol;Acc:HGNC:250]" | 9036 | -0.235 | -0.0776 | No |
| 35 | ADH1A | "alcohol dehydrogenase 1A (class I), alpha polypeptide [Source:HGNC Symbol;Acc:HGNC:249]" | 9283 | -0.254 | -0.0759 | No |
| 36 | UGT2A1 | UDP glucuronosyltransferase family 2 member A1 complex locus [Source:HGNC Symbol;Acc:HGNC:12542] | 9451 | -0.269 | -0.0668 | No |
| 37 | ADH6 | alcohol dehydrogenase 6 (class V) [Source:HGNC Symbol;Acc:HGNC:255] | 9504 | -0.274 | -0.0483 | No |
| 38 | RDH16 | retinol dehydrogenase 16 [Source:HGNC Symbol;Acc:HGNC:29674] | 9900 | -0.312 | -0.0535 | No |
| 39 | CYP3A4 | cytochrome P450 family 3 subfamily A member 4 [Source:HGNC Symbol;Acc:HGNC:2637] | 10193 | -0.340 | -0.0484 | No |
| 40 | CYP2A13 | cytochrome P450 family 2 subfamily A member 13 [Source:HGNC Symbol;Acc:HGNC:2608] | 10732 | -0.397 | -0.0577 | No |
| 41 | CYP26B1 | cytochrome P450 family 26 subfamily B member 1 [Source:HGNC Symbol;Acc:HGNC:20581] | 11022 | -0.433 | -0.0446 | No |
| 42 | CYP1A2 | cytochrome P450 family 1 subfamily A member 2 [Source:HGNC Symbol;Acc:HGNC:2596] | 11719 | -0.536 | -0.0548 | No |
| 43 | ALDH1A2 | aldehyde dehydrogenase 1 family member A2 [Source:HGNC Symbol;Acc:HGNC:15472] | 11796 | -0.548 | -0.0158 | No |
| 44 | CYP2C8 | cytochrome P450 family 2 subfamily C member 8 [Source:HGNC Symbol;Acc:HGNC:2622] | 11958 | -0.585 | 0.0197 | No |
| 45 | UGT2B15 | UDP glucuronosyltransferase family 2 member B15 [Source:HGNC Symbol;Acc:HGNC:12546] | 12225 | -0.652 | 0.0525 | No |
Table: GSEA details [plain text format]

  

Fig 2: KEGG\_RETINOL\_METABOLISM      
 Blue-Pink O' Gram in the Space of the Analyzed GeneSet

  

Fig 3: KEGG\_RETINOL\_METABOLISM: Random ES distribution      
 Gene set null distribution of ES for **KEGG\_RETINOL\_METABOLISM**
